# Cross sectional study on the prevalence and associated factors of iodine status in the population of Lausanne

**DOI:** 10.1038/s41598-025-24318-8

**Published:** 2025-11-18

**Authors:** Pauline Ducraux, Aurélien Thomas, Maïwenn Perrais, Julien Vaucher, Pedro Marques-Vidal

**Affiliations:** 1 Department of Medicine, Internal Medicine, Lausanne University Hospital (CHUV) and University of Lausanne, Rue du Bugnon 46, 1011 Lausanne, Switzerland; 2 Unit of Forensic Chemistry and Toxicology, University Centre of Legal Medicine Lausanne-Geneva, Geneva University Hospital and University of Geneva, Rue Michel-Servet 1, 1211 Geneva, Switzerland; 3 Faculty Unit of Toxicology, University Centre of Legal Medicine Lausanne-Geneva, Lausanne University Hospital and University of Lausanne, Chemin de La Vulliette 4, 1000 Lausanne, Switzerland; 4 Department of Internal Medicine and Specialties, Internal Medicine, Fribourg Hospital and University of Fribourg, Fribourg, Switzerland

**Keywords:** Iodine, Population study, Epidemiology, Deficiency, Endocrine system and metabolic diseases, Epidemiology

## Abstract

To assess iodine status and factors associated with iodine deficiency. Cross-sectional study conducted between 2003 and 2006 including 6,341 community dwellers, aged 35–75 years old, from a population-based sample from the city of Lausanne, Switzerland. Urinary iodine concentration (UIC) was used to assess iodine status. Iodine status was considered as adequate for UIC ≥ 100 mcg/L, mild deficiency if 50 ≤ UIC ≤ 99 mcg/L, moderate deficiency if 20 ≤ UIC ≤ 49 mcg/L, and severe deficiency if UIC < 20 mcg/L. A further categorization into adequate and deficient (UIC < 100 mcg/L) was performed. Overall, 70.5% of the participants presented with adequate iodine status, 23.0% with mild, 6.0% with moderate and 0.5% with severe iodine deficiency. After multivariable analysis, being a woman (odds ratio (95% confidence interval): 1.64 (1.45 ; 1.85)), hypertension 1.41 (1.24 ; 1.60) and increasing age (p for trend < 0.001) were associated with iodine deficiency. Conversely, increasing body mass index (p for trend < 0.001), current smoking 0.86 (0.75 ; 0.99) and thyroid hormone supplementation 0.48 (0.33 ; 0.70) were negatively associated with iodine deficiency. A positive correlation was found between UIC and urinary sodium concentration: 0.277, p < 0.001. In Lausanne, approximately one third of participants presented with iodine deficiency. Factors such as older age, being a woman or presenting with hypertension are associated with iodine deficiency, making those groups the main targets for further preventive interventions.

**Supplementary Information:**

The online version contains supplementary material available at 10.1038/s41598-025-24318-8.

## Introduction

Switzerland prominently features in medical history for introducing mandatory iodised salt regionally in 1922 and in the whole country in 1952^1^. Since then, iodine content of salt was increased progressively until 2014 where it stands at 25 mg/kg^1^. This public measure led to the rapid decrease in prevalence of goiter in the Swiss population^1^. Studies in different countries demonstrated similar results^2–5^. Several studies have shown that iodine fortification of salt is a safe strategy as well as an effective one to ensure a better iodine intake population-wide^3,4^. A worldwide overview showed a great increase of households covered with iodized salt after legislation for fortification^6^. Despite those encouraging results, less than half of European countries have mandatory iodized salt^7^.

The clinical and scientific assessment of an adequate iodine intake has evolved over time. Historically, the presence of goiter was used to define a deficiency. But this method has several limitations including the prolonged response time of this clinical marker after the normalization of iodine intake. Currently, ioduria is used to assess iodine intake as it was shown to be a reliable tool to estimate iodine intake^8–10^, with more than 90% of dietary iodine reflected in the urine^11,12^. Although spot urine sample shows a great variability on a day-to-day basis and is not recommended to assess an individual’s iodine status^9^, it is a reliable alternative to 24-h urine collection to estimate urinary iodine concentration (UIC) at a population level^13^.

Despite the recent improvements^14^, up to one third of the global population is considered to have an inadequate iodine intake^15^. Women, young or old adults, and people with a vegetarian/vegan diet are at higher risk of developing iodine deficiency^7,16,17^. Recent studies suggest that there is no significant increase in iodine intake in Swiss women and pregnant women since the last increase of salt iodination^18^. Also, besides gender and age, little is known regarding other possible determinants of low iodine status.

Hence, we used UIC from a large, population-based study conducted in Lausanne, Switzerland, to assess the iodine status and the factors associated with iodine deficiency. We hypothesized that women, elderly people, but also other socioeconomic factors would be associated with iodine deficiency as per UIC.

## Methods

### Participants and methods

#### Study design

Cross-sectional study conducted between 2003 and 2006 in a population-based sample from the city of Lausanne.

#### Participants

We used data from the baseline assessment of the CoLaus|PsyCoLaus study, a prospective, population-based study aimed at assessing the prevalence and determinants of cardiovascular disease in the city of Lausanne. The methodology of the CoLaus|PsyCoLaus study has been reported elsewhere^19^. Briefly, a single-step random sampling of the population aged 35 to 75 years at baseline living in the city of Lausanne (Switzerland) was conducted and a sample of 6,733 participants (participation rate 41%) was obtained. Participants were asked to attend the CoLaus|PsyCoLaus examination laboratory after an overnight fast. Pregnant women were not eligible for the study. Biological assays were performed by the CHUV Clinical Laboratory on fresh blood samples within 2 h of blood collection.

#### Inclusion and exclusion criteria

All participants of the initial CoLaus|PsyCoLaus Study Cohort were considered as eligible. Participants were excluded if they 1) lacked data on ioduria; 2) presented extreme values in UIC > 1 mg/L, or 3) had missing data of any covariate.

#### Iodine assessment

Spot urine samples were used to measure the participants’ UIC. Briefly, spot urine samples (200 µL) were diluted with 1.8 mL of HNO_3_ 1% solution containing 10 ng/mL Rhodium and 10 ng/mL Indium as internal standard^20,21^. Samples were analysed using an inductively coupled plasma mass spectrometer (ICP MS, 7800 Series, Agilent). The accuracy and precision of the method was routinely assessed using two commercial ClinCheck internal quality controls (http://www.recipe.de/en/index.html). Within the accreditation process, the method is also assessed 3 times a year with the QMEQAS external quality control of the Public Health Expertise and reference centre of Québec (www.inspq.qc.ca/ctqenglish/eqas/qmeqas/description).

UIC is considered a good indicator of a population iodine status and a reliable alternative to 24 h urine collection^13,22,23^. UIC adjusted for urinary creatinine was used to categorize participants in four categories according to Andersson et al^5^, except that no excess UIC category was created as it was not a targeted outcome. Participants were considered as having adequate iodine status if their UIC ≥ 100 μg/L, mild iodine deficiency if 50 ≤ IUC ≤ 99 μg/L, moderate iodine deficiency if 20 ≤ IUC ≤ 49 μg/L, and severe iodine deficiency if IUC < 20 μg/L^24^. A further categorization was conducted, dividing the participants into adequate and deficient (grouping mild, moderate, or severe deficiency) UIC. Finally, as the 100 μg/L has been established for children, another categorization was conducted considering adequate iodine status if UIC ≥ 60 μg/L as suggested by Zimmermann et al. (2012)^25^.

#### Other covariates

Other covariates were collected using self-filled questionnaires: gender; age; smoking status (never, former, current); presence of any type of diet, i.e. slimming, low-fat, low-carb, etc. (yes/no); marital status (living alone/living in couple); educational level (mandatory, apprenticeship, high school, and university), job type (high, middle, low, and not working), and alcohol consumption. Antihypertensive and antidiabetic drug treatment, dietary supplements, and thyroid hormone supplementation were assessed from the list of medicines provided by the participants.

Body weight and height were measured with participants barefoot and in light indoor clothes. Body weight was measured in kilograms to the nearest 100 g using a Seca® scale (Hamburg, Germany). Height was measured to the nearest 5 mm using a Seca® (Hamburg, Germany) height gauge. Body mass index (BMI) was calculated and categorized as underweight (BMI < 18 kg/m^2^), normal (18 ≤ BMI < 25 kg/m^2^), overweight (25 ≤ BMI < 30 kg/m^2^) and obese (BMI ≥ 30 kg/m^2^).

Blood pressure (BP) was measured thrice using an Omron® HEM-907 automated oscillometric sphygmomanometer after at least a 10-min rest in a seated position, and the average of the last two measurements was used. Hypertension was defined by a systolic BP (SBP) ≥ 140 mm Hg or a diastolic BP (DBP) ≥ 90 mm Hg or presence of antihypertensive drug treatment. Diabetes was defined by a fasting plasma glucose ≥ 7 mmol/l or presence of antidiabetic drug treatment.

Urinary creatinine was measured using the Jaffe isotope dilution mass spectrometry (IDMS) traceable measurement kinetic compensated method (Roche Diagnostics, Switzerland; coefficient of variation (CV) 2.9–0.7%). Urinary sodium was determined by indirect potentiometry (UniCEl DxC 800 Synchron System; Beckman Coulter, Brea, California, USA). No information regarding thyroid hormonal status was collected.

#### Statistical analysis

Statistical analyses were performed using Stata version 18.0 for windows® (Stata Corp, College Station, Texas, USA). Similar to Andersson et al. (2020)^18^, we computed the median UIC and its nonparametric 95% confidence interval by boostrapping (n = 1000) and also provided its interquartile range.

Analyses were conducted comparing participants with adequate to those with insufficient UIC for both thresholds (100 μg/L and 60 μg/L). Descriptive results were expressed as number of participants (percentage) for categorical variables and as average ± standard deviation or median [interquartile range] for continuous variables. Bivariate analyses were performed using chi-square or Fisher’s exact test for categorical variables and Student’s t-test or nonparametric Kruskal–Wallis test for continuous variables. Multivariable analysis of the factors associated with iodine deficiency were assessed by logistic regression, and the results were expressed as Odds ratio (OR) and 95% confidence interval (CI). The association between UIC and salt intake (expressed as spot urinary sodium concentration) was assessed by Spearman rank correlation analysis. Statistical significance was assessed for a two-sided test with p < 0.05.

## Results

### Characteristics of participants

**Fig. 1:**
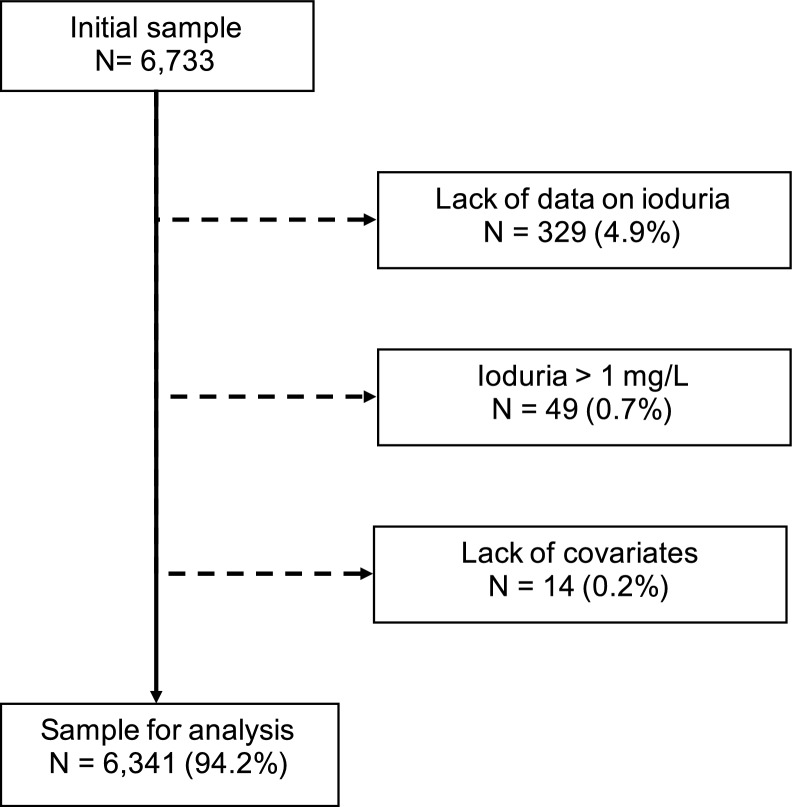
Flow diagram of participants.

Out of 6,733 participants, 329 were excluded due to lack of data on UIC (no urine available), 49 were excluded due to extreme values of UIC (> 1 mg/l) and 14 were not eligible because of missing covariates as shown on Fig. 1. The data of the remaining 6,341 participants were used for the main analysis. Characteristics of included and excluded participants are presented in Supplementary Table 1. Excluded participants were more likely to be older, not working and presented with hypertension more frequently. No difference was found between gender, educational level, or BMI. There was no pregnant woman in our data.

### Characteristics of participants according to category of ioduria

The median (95% CI) UIC was 131 (129–132) μg/L, while its interquartile range was [93—177]. The corresponding values for women and men were 123 [85—170] and 138 [102—183], respectively. Overall, the prevalence of adequate ioduria (≥ 100 μg/L) and of mild, moderate and severe iodine deficiency was 70.5%, 23.0%, 6.0% and 0.5%, respectively. The characteristics of the different iodine categories are summarized in Supplementary Table 2. Iodine deficiency had a higher frequency of women, older age, not working or hypertension. Conversely, adequate iodine status was associated with higher BMI, being a smoker and taking thyroid supplements.

Prevalence (95% CI) of deficient UIC was 29.5% (28.4–30.6) and 9.8% (9.1–10.6) for the 100 μg/L and the 60 μg/L thresholds, respectively. Table 1 shows the characteristics according to adequate or deficient UIC using the 100 μg/L threshold. Deficient UIC was associated with older age, higher frequencies of women, people living in couple, not working, never smokers, and hypertension, and a lower frequency of thyroid supplementation and a higher prevalence of menopaused women (Table 1). Similar findings were obtained when the 60 μg/L threshold was applied, except that no differences were found regarding marital and smoking status, and thyroid supplementation (Supplementary Table 3).

**Table 1:** Characteristics of the participants according to ioduria status using the 100 μg/L threshold, CoLaus|PsyCoLaus study, 2003–2006, Lausanne, Switzerland.

	**Adequate**	**Deficient**	**p-value**
Number	4470	1871	
Age (years)	51.8 ± 10.6	54.2 ± 10.7	< 0.001
Age categories (%)			< 0.001
[35–45]	1442 (32.2)	458 (24.5)	
[45–55]	1319 (29.5)	529 (28.3)	
[55–65]	1129 (25.3)	546 (29.2)	
[65–75]	580 (13.0)	338 (18.1)	
Women (%)	2200 (49.2)	1155 (61.7)	< 0.001
Living alone (%)	1434 (32.1)	661 (35.3)	0.012
Educational level (%)			0.198
University education	889 (19.9)	361 (19.3)	
High school	1057 (23.7)	484 (25.9)	
Apprenticeship	1568 (35.1)	657 (35.1)	
Mandatory education	956 (21.3)	369 (19.7)	
Job type (%)			< 0.001
High	563 (12.6)	191 (10.2)	
Middle	1806 (40.5)	723 (38.7)	
Low	914 (20.5)	325 (17.4)	
Not working	1178 (26.4)	630 (33.7)	
Smoking status (%)			0.011
Never	1772 (39.6)	799 (42.7)	
Former	1445 (32.4)	613 (32.8)	
Current	1253 (28.0)	459 (24.5)	
Alcohol consumption (%)			0.329
None	1236 (27.7)	560 (29.9)	
1–13/week	2448 (54.8)	997 (53.3)	
14–27/week	616 (13.8)	246 (13.2)	
28 +/week	170 (3.8)	68 (3.6)	
BMI (kg/m^2^)	25.9 ± 4.5	25.5 ± 4.5	< 0.001
BMI categories (%)			< 0.001
Underweight	60 (1.3)	41 (2.2)	
Normal	2017 (45.1)	936 (50.0)	
Overweight	1677 (37.6)	636 (34.0)	
Obese	716 (16.0)	258 (13.8)	
Hypertension (%)	1551 (34.7)	781 (41.8)	< 0.001
Diabetes (%)	288 (6.5)	122 (6.5)	0.903
Dietary supplements (%)	200 (6.0)	104 (7.1)	0.143
Thyroid supplementation (%)	129 (2.9)	37 (2.0)	0.039
Menopause (%) §	1106 (50.2)	682 (59.0)	< 0.001

BMI, body mass index. § women only. Results are expressed as number of participants (column percentage) for categorical variables and as average ± standard deviation for continuous variables. Between group comparisons performed using chi-square for categorical variables and student’s t-test for continuous variables. Deficient urinary iodine concentration defined as < 100 μg/L.

The results of the multivariable analysis of the factors associated with deficient UIC (< 100 μg/L) are summarized in Table 2. Increasing age, being a woman, or presenting with hypertension were significantly associated with a higher likelihood of presenting with deficient UIC, while current smoking, increasing BMI and thyroid supplementation were associated with a lower likelihood of presenting with deficient UIC. Similar findings were obtained when deficient UIC was defined by < 60 μg/L, except that increasing age and current smoking were no longer associated with deficiency (Table 2).

**Table 2:** Multivariable analysis of the factors associated with deficient UIC, as per ioduria < 100 or < 60 μg/L, CoLaus|PsyCoLaus study, 2003–2006, Lausanne, Switzerland.

	** < 100 μg/L**	**p-value**	** < 60 μg/L**	**p-value**
Age categories				
[35–45]	1 (ref.)		1 (ref.)	
[45–55]	1.23 (1.06; 1.43)	0.007	0.82 (0.65; 1.04)	0.101
[55–65]	1.39 (1.18; 1.63)	< 0.001	1.15 (0.91; 1.46)	0.255
[65–75]	1.54 (1.24; 1.90)	< 0.001	0.99 (0.72; 1.38)	0.974
P for trend, age categories	< 0.001		0.550	
Women vs. men	1.64 (1.45; 1.85)	< 0.001	2.33 (1.91; 2.84)	< 0.001
In couple vs. living alone	0.97 (0.86; 1.09)	0.600	1.04 (0.86; 1.24)	0.709
Job type				
High	1 (ref.)		1 (ref.)	
Middle	1.01 (0.83; 1.23)	0.907	0.95 (0.70; 1.28)	0.715
Low	1.01 (0.82; 1.25)	0.913	0.93 (0.66; 1.30)	0.669
Not working	1.09 (0.88; 1.36)	0.414	0.95 (0.68; 1.33)	0.780
Smoking status				
Never	1 (ref.)		1 (ref.)	
Former	0.99 (0.87; 1.13)	0.891	0.92 (0.75; 1.12)	0.388
Current	0.86 (0.75; 0.99)	0.041	0.91 (0.74; 1.13)	0.393
BMI categories				
Underweight	1.39 (0.92; 2.10)	0.119	1.28 (0.73; 2.26)	0.388
Normal	1 (ref.)		1 (ref.)	
Overweight	0.80 (0.71; 0.91)	0.001	0.73 (0.60; 0.89)	0.002
Obese	0.66 (0.55; 0.78)	< 0.001	0.63 (0.48; 0.83)	0.001
P for trend, BMI categories	< 0.001		0.010	
Hypertension (yes vs. no)	1.41 (1.24; 1.60)	< 0.001	1.55 (1.27; 1.89)	< 0.001
Thyroid supplementation (yes vs. no)	0.48 (0.33; 0.70)	< 0.001	0.39 (0.21; 0.76)	0.005

BMI, body mass index. Results are expressed as odds ratio and (95% confidence interval). Statistical analysis performed using logistic regression. Deficient urinary iodine concentration defined as < 100 or < 60 μg/L.

### Association between urinary sodium and iodine levels

The correlation between urinary sodium and urinary iodine levels was 0.277, p < 0.001. Urinary sodium concentrations were higher in men, with increasing BMI, among never smokers and participants with hypertension, and were lower with increasing age and in participants taking thyroid supplementation (Supplementary Table 4).

## Discussion

Our results indicate that iodine deficiency is present in approximately one third of middle-aged, community-dwelling people living in the city of Lausanne, Switzerland. Factors such as older age, being a woman or presenting with hypertension are associated with iodine deficiency, while higher BMI and smoking are associated with a lower likelihood of iodine deficiency.

### Prevalence of iodine deficiency

Median UIC were 131, 123 and 138 μg/L overall, for women and for men, respectively. Our results indicate that the iodine intake of the Swiss population is adequate, as the overall median is above the 100 μg/L threshold. Importantly, the values for women were higher than reported by Andersson et al. among women of reproductive age in 2020 (88 μg/L)^18^. Indeed, stratifying the analysis according to menopausal status showed a median UIC of 130 and 116 μg/L among non-menopaused and menopaused women, suggesting that the median UIC in Swiss women has decreased.

As previously shown, UIC is considered a reliable tool to estimate iodine status at a population level^9–11,13^, even though its day-to-day variability prevents it from assessing an indivual’s iodine status^9^. Iodine status represent a challenge for public health as even mild to moderate iodine deficiency can lead to toxic nodular goiter and hyperthyroidism^26^. Monitoring UIC in order to improve iodine intake is a major component of public measures against thyroid disorders.

In this study, one third to one tenth of participants presented with deficient ioduria, depending on the threshold applied; of those, mostly presented with a mild deficiency. A study conducted on 24-h urine between 2009 and 2013 by Stalder et al.^16^ using the 100 μg/L threshold reported a prevalence of inadequate iodine intake, defined as an estimated average requirement of 95 μg/day, at 14% for women and 4% for men, values lower than reported in this study.

Altogether, Swiss households seem to be well covered in iodized salt^18^. Iodized salt represents 44% of the iodine intake of the Swiss population^27^, and a positive correlation was found between urinary sodium and iodine in this study, suggesting that still a sizable fraction of iodine intake comes from iodized salt. Still, dietary habits tend to evolve toward more processed foods and other sources of salt that are not necessarily iodized^28^. Iodized salt is more expensive than non-iodized salt, which might explain its decrease in consumption. Indeed, iodized salt contributed only to 24% of total salt intake in the Italian adult population^2^ and, in this study, participants with iodine deficiency consumed more salt than those with adequate iodine status, suggesting consumption of non-iodized salt. Besides, the decrease of salt consumption in response to prevention campaigns against hypertension represents a new challenge for iodine fortification^7^. As the consumption of salt and iodine intake are closely related, the reduction of cardiovascular risks and thyroid disorders must be considered jointly^2,24^. Indeed, a salt intake below 5 g/day as recommended by the WHO will likely fail to achieve adequate iodine intake^2^. Further, as salt fortification is not compulsory in Switzerland, alternate ways to provide an adequate supply of iodine to the Swiss population must be considered, and some authors suggested the mandatory use of fortified salt in all processed foods^2,18^.

### Factors associated with iodine deficiency

One third of women presented with iodine deficiency as defined by UIC < 100 μg/L, and approximately one in seven (13.0%) by UIC < 60 μg/L. The first finding is in agreement with the literature^27,29^, and could be explained by lower food intake and lesser use of salt by women relative to men^2,30^, although this information is lacking in this study.

The risk of iodine deficiency as defined by UIC < 100 μg/L increased with age, a finding in agreement with one study^31^ but not with another^16^. One possible explanation are changes in dietary intake associated with recommendations to decrease salt consumption due to increased prevalence of hypertension, as urinary sodium concentrations were lower in older participants.

Participants with hypertension presented more frequently with iodine deficiency, a finding also reported elsewhere^32^. As participants with hypertension presented a higher mean urinary sodium concentration, the initial hypothesis of restricted salt consumption in this group was challenged. Indeed, a previous study reported that only 8% of people with hypertension report consuming a low-salt diet^33^, and the Swiss Survey of Salt reported no difference regarding hypertension prevalence between people eating less or above 5 g of salt daily^34^. Two studies reported that sodium intake and sodium restriction in healthy populations and in participants with hypertension does not lower iodine levels^35,36^, while another study found a significant increase of iodine deficiency associated with salt restriction in women^37^. Overall, the associations between urinary sodium and iodine deficiency should be further explored.

Higher BMI was a protective factor against iodine deficiency^2,38,39^. Again, the most likely explanation is an increased food intake by people with obesity, including an increased consumption of iodine sources, including salt. Indeed, the recent Swiss Survey of Salt reported that people with normal BMI had a lower salt consumption than people with overweight or obesity^34^.

Surprisingly, smoking was associated with lower risk of deficient UIC. Our findings contradict results from previous studies that reported clear associations between smoking status and iodine intake^40,41^. This could be due to a higher proportion of older participants and especially older women among non-smokers^42^, or an increased salt consumption by smokers^43^. Still, urinary sodium levels were lower among current smokers than nonsmokers, suggesting that other mechanisms or behaviors might occur.

### Implication for clinical practice

In Switzerland, the last increase in iodine concentration of salt occurred in 2014 but did not improve iodine coverage in the Swiss population^18^. Also, given the recommendations to decrease salt intake for cardiovascular prevention, it seems unlikely that a new increase of iodine concentration in salt would allow for a sufficient iodoprophylaxis in the Swiss population. A possible alternative would be to mandate salt iodization of processed foods, although this might require changes in food processing or legislation.

### Strengths and limitations

The large sample size constitutes a major strength of this study and can be considered representative of the Lausanne population to a certain extend. UIC is a method of choice to assess iodine status at the population level, as it is less demanding and a reliable alternative to 24 h urine collection. An additional strength is the number of covariates, which allowed for a wide range of analyses and adjustments.

This study also has some limitations. First, it was conducted in a single geographical location, and results might not be generalizable to other settings in Switzerland, as it has been shown that dietary intake varies by linguistic region^44^. Second, although it is commonly acknowledged that 90% of dietary iodine intake is excreted in urine, this pourcentage could vary with iodine status. Still, we believe that our results remain relevant, particularly for comparison with other studies using UIC. Third, no information regarding thyroid status was collected. Hence, we could not associate the UIC with thyroid disease, and it would be important that future studies perform such analyses. Finally, the data was collected for period 2003–2006, which is relatively old, and changes might have occurred as the dietary intake of the population has improved^45^. Still, to our knowledge, no recent study assessed the UIC of the Swiss population. More recent studies on salt intake conducted in Switzerland suggest that in both children^46^ and adults^34^ have a high intake of salt, but whether this salt contains added iodine was not assessed. It would be important that the iodine status of the Swiss population be evaluated using more recent information.

## Conclusion

Approximately one third of middle-aged, community-dwelling people living in the city of Lausanne, Switzerland presented with iodine deficiency. Factors such as older age, being a woman or presenting with hypertension are associated with deficient UIC, making those groups priority targets for preventive measures.

## Supplementary Information

Supplementary Information.

## Data Availability

The datasets used and analysed during the current study are available on reasonable request at research.colaus@chuv.ch.

## References

[1] ASSM. Bulletin Suisse ASSM 2022. 2022.

[2] Iacone, R. et al. Iodine intake from food and iodized salt as related to dietary salt consumption in the italian adult general population. *Nutrients* **13**(10), 3486 (2021). 34684487 10.3390/nu13103486 PMC8537510

[3] Dold, S. et al. Universal salt iodization provides sufficient dietary iodine to achieve adequate iodine nutrition during the first 1000 days: A cross-sectional multicenter study. *J. Nutr.* **148**(4), 587–598 (2018). 29659964 10.1093/jn/nxy015

[4] Li, Y. et al. Efficacy and safety of long-term universal salt iodization on thyroid disorders: Epidemiological evidence from 31 provinces of Mainland China. *Thyroid* **30**(4), 568–579 (2020). 32075540 10.1089/thy.2019.0067

[5] Andersson, M., Karumbunathan, V. & Zimmermann, M. B. Global iodine status in 2011 and trends over the past decade. *J. Nutr.* **142**(4), 744–750 (2012). 22378324 10.3945/jn.111.149393

[6] Andersson, M., de Benoist, B. & Rogers, L. Epidemiology of iodine deficiency: Salt iodisation and iodine status. *Best Pract. Res. Clin. Endocrinol. Metab.* **24**(1), 1–11 (2010). 20172466 10.1016/j.beem.2009.08.005

[7] Bath, S. C. et al. A systematic review of iodine intake in children, adults, and pregnant women in Europe-comparison against dietary recommendations and evaluation of dietary iodine sources. *Nutr. Rev.* **80**(11), 2154–2177 (2022). 35713524 10.1093/nutrit/nuac032 PMC9549594

[8] Zhao, W. et al. Prevalence of goiter and thyroid nodules before and after implementation of the universal salt iodization program in mainland China from 1985 to 2014: A systematic review and meta-analysis. *PLoS ONE* **9**(10), e109549 (2014). 25313993 10.1371/journal.pone.0109549 PMC4196906

[9] Pearce, E. N. & Caldwell, K. L. Urinary iodine, thyroid function, and thyroglobulin as biomarkers of iodine status. *Am. J. Clin. Nutr.* **104**(3), 898s–901s (2016). 27534636 10.3945/ajcn.115.110395 PMC5004493

[10] Organization WH. Assessment of iodine deficiency disorders and monitoring their elimination : a guide for programme managers. – 3rd ed.; 2007.

[11] Zimmermann, M. B. Iodine deficiency. *Endocr Rev.* **30**(4), 376–408 (2009). 19460960 10.1210/er.2009-0011

[12] Vought, R. L. & London, W. T. Iodine intake, excretion and thyroidal accumulation in healthy subjects. *J. Clin. Endocrinol. Metab.* **27**(7), 913–919 (1967). 4165697 10.1210/jcem-27-7-913

[13] König, F., Andersson, M., Hotz, K., Aeberli, I. & Zimmermann, M. B. Ten repeat collections for urinary iodine from spot samples or 24-hour samples are needed to reliably estimate individual iodine status in women. *J. Nutr.* **141**(11), 2049–2054 (2011). 21918061 10.3945/jn.111.144071

[14] Zimmermann, M. B. & Andersson, M. Prevalence of iodine deficiency in Europe in 2010. *Ann. Endocrinol. (Paris)* **72**(2), 164–166 (2011). 21511244 10.1016/j.ando.2011.03.023

[15] Zimmermann, M. B. & Andersson, M. Update on iodine status worldwide. *Curr. Opin. Endocrinol. Diabetes Obes.* **19**(5), 382–387 (2012). 22892867 10.1097/MED.0b013e328357271a

[16] Stalder, E. et al. Use of day and night urinary iodine excretion to estimate the prevalence of inadequate iodine intakes via the estimated average requirement cut-point method. *Swiss Med. Wkly.* **149**, w20090 (2019). 31154659 10.4414/smw.2019.20090

[17] Eveleigh, E. R., Coneyworth, L. J., Avery, A. & Welham, S. J. M. Vegans, vegetarians, and omnivores: How does dietary choice influence iodine intake? a systematic review. *Nutrients* **12**(6), 1606 (2020). 32486114 10.3390/nu12061606 PMC7352501

[18] Andersson, M., Hunziker, S., Fingerhut, R., Zimmermann, M. B. & Herter-Aeberli, I. Effectiveness of increased salt iodine concentration on iodine status: Trend analysis of cross-sectional national studies in Switzerland. *Eur. J. Nutr.* **59**(2), 581–593 (2020). 30843107 10.1007/s00394-019-01927-4

[19] Firmann, M. et al. The CoLaus study: A population-based study to investigate the epidemiology and genetic determinants of cardiovascular risk factors and metabolic syndrome. *BMC Cardiovasc. Disord.* **8**, 6 (2008). 18366642 10.1186/1471-2261-8-6 PMC2311269

[20] Jafari, P. et al. Trace element intakes should be revisited in burn nutrition protocols: A cohort study. *Clin. Nutr.* **37**(3), 958–964 (2018). 28455105 10.1016/j.clnu.2017.03.028

[21] Zhang, Z. Y. et al. Ambulatory blood pressure in relation to plasma and urinary manganese. *Hypertension* **75**(4), 1133–1139 (2020). 32114854 10.1161/HYPERTENSIONAHA.119.13649

[22] Hatch-McChesney, A. & Lieberman, H. R. Iodine and iodine deficiency: A comprehensive review of a Re-Emerging issue. *Nutrients* **14**(17), 3474 (2022). 36079737 10.3390/nu14173474 PMC9459956

[23] Rohner, F. et al. Biomarkers of nutrition for development–iodine review. *J. Nutr.* **144**(8), 1322s-s1342 (2014). 24966410 10.3945/jn.113.181974 PMC4093988

[24] WHO. Assessment of iodine deficiency disorders and monitoring their elimination : a guide for programme managers. – 3rd ed. 2007.

[25] Zimmermann, M. B. & Andersson, M. Assessment of iodine nutrition in populations: Past, present, and future. *Nutr. Rev.* **70**(10), 553–570 (2012). 23035804 10.1111/j.1753-4887.2012.00528.x

[26] Zimmermann, M. B. & Boelaert, K. Iodine deficiency and thyroid disorders. *Lancet Diabetes Endocrinol.* **3**(4), 286–295 (2015). 25591468 10.1016/S2213-8587(14)70225-6

[27] Haldimann, M., Bochud, M., Burnier, M., Paccaud, F. & Dudler, V. Prevalence of iodine inadequacy in Switzerland assessed by the estimated average requirement cut-point method in relation to the impact of iodized salt. *Pub. Health Nutr.* **18**(8), 1333–1342 (2015). 25231207 10.1017/S1368980014002018 PMC10271515

[28] Zimmermann, M. B. & Andersson, M. GLOBAL ENDOCRINOLOGY: Global perspectives in endocrinology: Coverage of iodized salt programs and iodine status in 2020. *Eur. J. Endocrinol.* **185**(1), R13-r21 (2021). 33989173 10.1530/EJE-21-0171 PMC8240726

[29] Han, X., Ding, S., Lu, J. & Li, Y. Global, regional, and national burdens of common micronutrient deficiencies from 1990 to 2019: A secondary trend analysis based on the Global Burden of Disease 2019 study. *EClinicalMedicine* **44**, 101299 (2022). 35198923 10.1016/j.eclinm.2022.101299 PMC8850322

[30] Vandevijvere, S. et al. Urinary sodium and iodine concentrations among Belgian adults: Results from the first national health examination survey. *Eur. J. Clin. Nutr.* **75**(4), 689–696 (2021). 33033379 10.1038/s41430-020-00766-5

[31] Laurberg, P., Andersen, S., Bülow Pedersen, I. & Carlé, A. Hypothyroidism in the elderly: Pathophysiology, diagnosis and treatment. *Drugs Aging.* **22**(1), 23–38 (2005). 15663347 10.2165/00002512-200522010-00002

[32] Menon, V. U. et al. Iodine status and its correlations with age, blood pressure, and thyroid volume in South Indian women above 35 years of age (Amrita Thyroid Survey). *Indian J. Endocrinol. Metab.* **15**(4), 309–315 (2011). 22029002 10.4103/2230-8210.85584 PMC3193780

[33] Grange, M. et al. Lost in translation: Dietary management of cardiovascular risk factors is seldom implemented. *Prev. Med.* **76**, 68–73 (2015). 25895841 10.1016/j.ypmed.2015.03.024

[34] Burnier, M., Paccaud, F. M. & Bochud, M. Clinical profiles and factors associated with a low sodium intake in the population: An analysis of the swiss survey on salt. *Nutrients* **12**(11), 3591 (2020). 33238516 10.3390/nu12113591 PMC7700385

[35] Charlton, K. E., Jooste, P. L., Steyn, K., Levitt, N. S. & Ghosh, A. A lowered salt intake does not compromise iodine status in Cape Town, South Africa, where salt iodization is mandatory. *Nutrition* **29**(4), 630–634 (2013). 23274097 10.1016/j.nut.2012.09.010

[36] Musso, N. et al. Low-Salt intake suggestions in hypertensive patients do not jeopardize urinary iodine excretion. *Nutrients* **10**(10), 1548 (2018). 30347728 10.3390/nu10101548 PMC6213341

[37] Tayie, F. A. & Jourdan, K. Hypertension, dietary salt restriction, and iodine deficiency among adults. *Am. J. Hypertens.* **23**(10), 1095–1102 (2010). 20559287 10.1038/ajh.2010.120

[38] Jin, M. et al. U-Shaped associations between urinary iodine concentration and the prevalence of metabolic disorders: A cross-sectional study. *Thyroid* **30**(7), 1053–1065 (2020). 32188373 10.1089/thy.2019.0516

[39] Shen, X. et al. Associations between urinary iodine concentration and the prevalence of metabolic disorders: A cross-sectional study. *Front. Endocrinol. (Lausanne)* **14**, 1153462 (2023). 37223035 10.3389/fendo.2023.1153462 PMC10200914

[40] Ozpinar, A. et al. Iodine status in turkish populations and exposure to iodide uptake inhibitors. *PLoS ONE* **9**(2), e88206 (2014). 24505430 10.1371/journal.pone.0088206 PMC3914924

[41] Cho, N. H. et al. Interaction between cigarette smoking and iodine intake and their impact on thyroid function. *Clin. Endocrinol. (Oxf)* **73**(2), 264–270 (2010). 20105185 10.1111/j.1365-2265.2010.03790.x

[42] OFS. Enquête suisse sur la santé (ESS) 2017 - Santé et genre 2020

[43] Ma, J. & Lee, Y. K. Examining the association between cigarette smoking quantity and subjective salt taste preference and salt-related eating behavior. *Korean J. Fam. Med.* **44**(6), 335–341 (2023). 37647943 10.4082/kjfm.23.0027 PMC10667072

[44] Chatelan, A. et al. Major differences in diet across three linguistic regions of Switzerland: Results from the first national nutrition survey menuCH. *Nutrients* **9**(11), 1163 (2017). 29068399 10.3390/nu9111163 PMC5707635

[45] Marques-Vidal, P., Gaspoz, J. M., Theler, J. M. & Guessous, I. Twenty-year trends in dietary patterns in French-speaking Switzerland: Toward healthier eating. *Am. J. Clin. Nutr.* **106**(1), 217–224 (2017). 28592598 10.3945/ajcn.116.144998

[46] Rios-Leyvraz, M. et al. Estimation of salt intake and excretion in children in one region of Switzerland: A cross-sectional study. *Eur. J. Nutr.* **58**(7), 2921–2928 (2019). 30341681 10.1007/s00394-018-1845-4

