# Supplementary material for: Cross sectional study on the prevalence and associated factors of iodine status in the population of Lausanne
Source: Sci Rep. 2025 Nov 18;15:40493. doi: 10.1038/s41598-025-24318-8 (PMC12627634; doi:10.1038/s41598-025-24318-8)
Supplement: Supplementary file 1 — Supplementary Information. [file 41598_2025_24318_MOESM1_ESM.docx]

**Supplementary information**

**Supplementary table 1**: characteristics of included and excluded participants, CoLaus|PsyCoLaus study, 2003-2006, Lausanne, Switzerland.

|  | **Included** | **Excluded** | **p-value** |
| --- | --- | --- | --- |
| Number | 6341 | 392 |  |
| Age (years) | 52.5 ± 10.7 | 54.7 ± 11.1 | <0.001 |
| Women (%) | 3355 (52.9) | 189 (48.2) | 0.071 |
| Living alone (%) | 2095 (33.0) | 119 (30.8) | 0.369 |
| Educational level (%) |  |  | 0.248 |
| University | 1250 (19.7) | 70 (18.5) |  |
| High school | 1541 (24.3) | 84 (22.2) |  |
| Apprenticeship | 2225 (35.1) | 152 (40.2) |  |
| Mandatory education | 1325 (20.9) | 72 (19.1) |  |
| Job type (%) |  |  | 0.019 |
| High | 754 (11.9) | 49 (12.7) |  |
| Middle | 2529 (40.0) | 133 (34.5) |  |
| Low | 1239 (19.5) | 67 (17.4) |  |
| Not working | 1808 (28.6) | 137 (35.5) |  |
| Smoking status (%) |  |  | 0.868 |
| Never | 2571 (40.5) | 161 (41.7) |  |
| Former | 2058 (32.5) | 125 (32.4) |  |
| Current | 1712 (27.0) | 100 (25.9) |  |
| Alcohol consumption (%) |  |  | 0.856 |
| None | 1796 (28.3) | 119 (30.4) |  |
| 1-13/week | 3445 (54.3) | 208 (53.1) |  |
| 14-27/week | 862 (13.6) | 51 (13.0) |  |
| 28+/week | 238 (3.8) | 14 (3.6) |  |
| BMI (kg/m^2^) | 25.8 ± 4.5 | 26.0 ± 4.5 | 0.321 |
| BMI categories (%) |  |  | 0.876 |
| Underweight | 101 (1.6) | 7 (1.8) |  |
| Normal | 2953 (46.6) | 176 (45.2) |  |
| Overweight | 2313 (36.5) | 149 (38.3) |  |
| Obese | 974 (15.4) | 57 (14.7) |  |
| Kidney function (eGFR) |  |  |  |
| Hypertension (%) | 2332 (36.8) | 168 (43.2) | 0.011 |
| Diabetes (%) | 410 (6.5) | 26 (6.7) | 0.841 |

BMI, body mass index. Results are expressed as number of participants (column percentage) for categorical variables and as average ± standard deviation for continuous variables. Between group comparisons performed using chi-square for categorical variables and student’s t-test for continuous variables.

**Supplementary table 2**: characteristics of participants according to categories of urinary iodine concentration, CoLaus|PsyCoLaus study, 2003-2006, Lausanne, Switzerland.

|  | **≥100 μg/L** | **50-99 μg/L** | **20-49 μg/L** | **<20 μg/L** | **p-value** |
| --- | --- | --- | --- | --- | --- |
| Number | 4470 | 1460 | 382 | 29 |  |
| Age (years) | 51.8 ± 10.6 | 54.4 ± 10.8 | 53.3 ± 10.6 | 54.2 ± 11.8 | <0.001 |
| Age categories (%) |  |  |  |  | <0.001 |
| [35-45[ | 1442 (32.3) | 344 (23.6) | 105 (27.5) | 9 (31.0) |  |
| [45-55[ | 1319 (29.5) | 418 (28.6) | 104 (27.2) | 7 (24.1) |  |
| [55-65[ | 1129 (25.3) | 421 (28.8) | 118 (30.9) | 7 (24.1) |  |
| [65-75[ | 580 (13.0) | 277 (19.0) | 55 (14.4) | 6 (20.7) |  |
| Gender (%) |  |  |  |  | <0.001 |
| Women | 2200 (49.2) | 855 (58.6) | 279 (73.0) | 21 (72.4) |  |
| Men | 2270 (50.8) | 605 (41.4) | 103 (27.0) | 8 (27.6) |  |
| Marital status (%) |  |  |  |  | 0.077 |
| Single | 1434 (32.1) | 512 (35.1) | 137 (35.9) | 12 (41.4) |  |
| In couple | 3035 (67.9) | 948 (64.9) | 245 (64.1) | 17 (58.6) |  |
| Educational level (%) |  |  |  |  | 0.337 |
| University | 889 (19.9) | 287 (19.7) | 69 (18.1) | 5 (17.2) |  |
| High school | 1057 (23.7) | 362 (24.8) | 114 (29.8) | 8 (27.6) |  |
| Apprenticeship | 1568 (35.1) | 514 (35.2) | 131 (34.3) | 12 (41.4) |  |
| Mandatory education | 956 (21.4) | 297 (20.3) | 68 (17.8) | 4 (13.8) |  |
| Job type (%) |  |  |  |  | <0.001 |
| High | 563 (12.6) | 152 (10.4) | 35 (9.2) | 4 (13.8) |  |
| Middle | 1806 (40.5) | 550 (37.7) | 160 (42.0) | 13 (44.8) |  |
| Low | 914 (20.5) | 260 (17.8) | 62 (16.3) | 3 (10.3) |  |
| Not working | 1178 (26.4) | 497 (34.1) | 124 (32.6) | 9 (31.0) |  |
| Smoking status (%) |  |  |  |  | 0.011 |
| Never | 1772 (39.6) | 606 (41.5) | 179 (46.9) | 14 (48.3) |  |
| Former | 1445 (32.3) | 499 (34.2) | 104 (27.2) | 10 (34.5) |  |
| Current | 1253 (28.0) | 355 (24.3) | 99 (25.9) | 5 (17.2) |  |
| Alcohol consumption (%) |  |  |  |  | ‡ 0.252 |
| None | 1236 (27.7) | 432 (29.6) | 119 (31.2) | 9 (31.0) |  |
| 1-13/week | 2448 (54.8) | 766 (52.5) | 215 (56.3) | 16 (55.2) |  |
| 14-27/week | 616 (13.8) | 207 (14.2) | 37 (9.7) | 2 (6.9) |  |
| 28+/week | 170 (3.8) | 55 (3.8) | 11 (2.9) | 2 (6.9) |  |
| BMI (kg/m^2^) | 25.9 ± 4.5 | 25.6 ± 4.6 | 24.9 ± 4.2 | 23.7 ± 3.7 | <0.001 |
| BMI categories (%) |  |  |  |  | ‡ <0.001 |
| Underweight | 60 (1.3) | 30 (2.1) | 10 (2.6) | 1 (3.5) |  |
| Normal | 2017 (45.1) | 703 (48.2) | 212 (55.5) | 21 (72.4) |  |
| Overweight | 1677 (37.5) | 516 (35.3) | 115 (30.1) | 5 (17.2) |  |
| Obese | 716 (16.0) | 211 (14.5) | 45 (11.8) | 2 (6.9) |  |
| Kidney function (eGFR) |  |  |  |  |  |
| Hypertension (%) |  |  |  |  | <0.001 |
| No | 2917 (65.3) | 844 (57.9) | 228 (59.7) | 16 (55.2) |  |
| Yes | 1551 (34.7) | 614 (42.1) | 154 (40.3) | 13 (44.8) |  |
| Diabetes (%) |  |  |  |  | ‡ 0.381 |
| No | 4176 (93.6) | 1357 (93.1) | 359 (94.5) | 29 (100) |  |
| Yes | 288 (6.5) | 101 (6.9) | 21 (5.5) | 0 (0) |  |
| Dietary supplements (%) |  |  |  |  | ‡ 0.207 |
| No | 3132 (94.0) | 1051 (92.4) | 282 (94.6) | 23 (95.8) |  |
| Yes | 200 (6.0) | 87 (7.6) | 16 (5.4) | 1 (4.2) |  |
| Thyroid supplementation (%) |  |  |  |  | ‡ 0.069 |
| No | 4341 (97.1) | 1427 (97.7) | 379 (99.2) | 28 (96.6) |  |
| Yes | 129 (2.9) | 33 (2.3) | 3 (0.8) | 1 (3.5) |  |
| Menopause (%) § |  |  |  |  | <0.001 |
| No | 1097 (49.8) | 336 (39.3) | 127 (45.5) | 10 (47.6) |  |
| Yes | 1106 (50.2) | 519 (60.7) | 152 (54.5) | 11 (52.4) |  |

BMI, body mass index. § women only; ‡ result questionable. Results are expressed as number of participants (column percentage) for categorical variables and as average ± standard deviation for continuous variables. Between group comparisons performed using chi-square for categorical variables and analysis of variance for continuous variables.

**Supplementary table 3**: characteristics of the participants according to ioduria status using the 60 μg/L threshold, CoLaus|PsyCoLaus study, 2003-2006, Lausanne, Switzerland.

|  | **Adequate** | **Deficient** | **p-value** |
| --- | --- | --- | --- |
| Number | **5,720** | **621** |  |
| Age (years) | 52.4 ± 10.7 | 53.6 ± 10.9 | 0.006 |
| Age categories (%) |  |  | 0.004 |
| [35-45[ | 1,723 (30.1) | 177 (28.5) |  |
| [45-55[ | 1,697 (29.7) | 151 (24.3) |  |
| [55-65[ | 1,481 (25.9) | 194 (31.2) |  |
| [65-75[ | 819 (14.3) | 99 (15.9) |  |
| Women (%) | 2917 (51.0) | 438 (70.5) | <0.001 |
| Living alone (%) | 1872 (32.7) | 224 (36.1) | 0.091 |
| Educational level (%) |  |  | 0.50 |
| University education | 1,128 (19.7) | 122 (19.6) |  |
| High school | 1,375 (24.0) | 166 (26.7) |  |
| Apprenticeship | 2,015 (35.2) | 210 (33.8) |  |
| Mandatory education | 1,202 (21.0) | 123 (19.8) |  |
| Job type (%) |  |  | 0.018 |
| High | 692 (12.1) | 62 (10.0) |  |
| Middle | 2,281 (39.9) | 248 (40.0) |  |
| Low | 1,135 (19.9) | 104 (16.8) |  |
| Not working | 1,602 (28.1) | 206 (33.2) |  |
| Smoking status (%) |  |  | 0.053 |
| Never | 2,291 (40.1) | 280 (45.1) |  |
| Former | 1,874 (32.8) | 184 (29.6) |  |
| Current | 1,555 (27.2) | 157 (25.3) |  |
| Alcohol consumption (%) |  |  | 0.12 |
| None | 1,608 (28.1) | 188 (30.3) |  |
| 1-13/week | 3,100 (54.2) | 345 (55.6) |  |
| 14-27/week | 796 (13.9) | 66 (10.6) |  |
| 28+/week | 216 (3.8) | 22 (3.5) |  |
| BMI (kg/m^2^) | 25.9 ± 4.5 | 25.1± 4.3 | <0.001 |
| BMI categories (%) |  |  | <0.001 |
| Underweight | 86 (1.5) | 15 (2.4) |  |
| Normal | 2,615 (45.7) | 338 (54.4) |  |
| Overweight | 2,125 (37.2) | 188 (30.3) |  |
| Obese | 894 (15.6) | 80 (12.9) |  |
| Hypertension (%) | 2,073 (36.3) | 259 (41.7) | 0.008 |
| Diabetes (%) | 377 (6.6) | 33 (5.3) | 0.22 |
| Dietary supplements (%) | 272 (6.3) | 32 (6.6) | 0.81 |
| Thyroid supplementation (%) | 156 (2.7) | 10 (1.6) | 0.098 |
| Menopause (%) § | 1,542 (52.8) | 246 (56.2) | 0.19 |

BMI, body mass index. § women only. Results are expressed as number of participants (column percentage) for categorical variables and as average ± standard deviation for continuous variables. Between group comparisons performed using chi-square for categorical variables and student’s t-test for continuous variables. Deficient urinary iodine concentration defined as <60 μg/L.

**Supplementary table 4**: urinary sodium concentrations according to some characteristics of the participants, CoLaus|PsyCoLaus study, 2003-2006, Lausanne, Switzerland.

|  | **Sodium (mmol/L)** | **p-value** |
| --- | --- | --- |
| Age categories (%) |  | <0.001 |
| [35-45[ | 117 [82 - 156] |  |
| [45-55[ | 112 [79 - 148] |  |
| [55-65[ | 108 [74 - 143] |  |
| [65-75[ | 107 [75 - 139] |  |
| Gender (%) |  | <0.001 |
| Women | 101 [69 - 135] |  |
| Men | 125 [90 - 159] |  |
| Smoking status (%) |  | <0.001 |
| Never | 114 [80 - 152] |  |
| Former | 112 [81 - 148] |  |
| Current | 106 [70 - 143] |  |
| BMI categories (%) |  | <0.001 |
| Underweight | 86 [56 - 121] |  |
| Normal | 105 [72 - 139] |  |
| Overweight | 117 [83 - 154] |  |
| Obese | 124 [87 - 159] |  |
| Hypertension (%) |  | 0.038 |
| No | 110 [76 - 146] |  |
| Yes | 114 [80 - 150] |  |
| Thyroid supplementation (%) |  | 0.028 |
| No | 112 [78 - 148] |  |
| Yes | 102 [64 - 143] |  |

BMI, body mass index. Results are as median [interquartile range]. Between group comparisons performed using Kruskal-Wallis nonparametric test.
